# On the Juridical Relevance of the Phenomenological Notion of Person in Max Scheler and Edith Stein

**DOI:** 10.1007/s11196-021-09823-z

**Published:** 2021-02-26

**Authors:** Francesco Galofaro

**Affiliations:** Philosophy and Science of Education Dep., University of Turin, Turin, Italy

**Keywords:** Socio-semiotics, Values, Community, Mass, Human rights, Exclusion, Responsibility, Voluntary termination of pregnancy, Artificial intelligence, Electronic personality

## Abstract

The paper presents a semiotic interpretation of the phenomenological debate on the notion of person, focusing in particular on Edmund Husserl, Max Scheler, and Edith Stein. The semiotic interpretation lets us identify the categories that orient the debate: collective/individual and subject/object. As we will see, the phenomenological analysis of the relation between person and social units such as the community, the association, and the mass shows similarities to contemporary socio-semiotic models. The difference between community, association, and mass provides an explanation for the establishment of legal systems. The notion of person we inherit from phenomenology can also be useful in facing juridical problems raised by the use of non-human decision-makers such as machine learning algorithms and artificial intelligence applications.

## Introduction

The phenomenological definition of “person” was proposed by Max Scheler [1] in the framework of a scientific study of ethics aimed at going beyond the limit of Kant’s formalistic point of view on morals, thanks to the phenomenological methods proposed by Edmund Husserl [2]. This issue is still much debated in philosophy of law: formalistic points of view, such as legal positivism, seem too weak, since they justify every internally coherent legal system, including the Nuremberg Laws. On the other hand, material approaches do not explain cultural variations. Alan Dershowitz provided a good review of different unsatisfactory philosophical approaches [3]. Unfortunately, his solution, according to which legal systems empirically emerge from our collective experience of injustice, only postpones the problem: is there such a thing as “collective experience”? As we will see, on the basis of Max Scheler’s personalism, Edith Stein was to advance a phenomenological analysis of this subject, which is relevant from a semiotic point of view because it involves technical notions, such as collective actor and modal value, which allow us to glimpse a strong connection between formation of law and meaning. In fact, what will emerge is that Scheler’s analysis of ethics expresses a relation between *values* embodied by *objects* targeted by *acts* of which the *subject* is the *person*. In particular, the person is given only where it possesses a *power to do* by means of the body [1]. It is almost inevitable to interpret this definition in semiotic terms: in particular, we consider Scheler’s notion of person as an anthropomorphic actor who embodies the actantial function of a qualified subject [4]. It will become apparent that the person in Scheler’s definition cannot be identified with other kinds of actor individuated by Greimas’ typology.

### What a Person is Not: Reason and Memory

According to Scheler, the notion of person cannot be reduced to “rationality” [1: 371]. The rules of rational, logical reasoning leading to universal conclusions must be shared by all rational humans. In a similar way, different Greek philosophers argue that there is only one general intellect, the νοῦς.^1^ We find a similar conclusion in Scholastic philosophy, according to which intellect is a general form^2^: the unity of reason would lead to the conclusion that there is only one collective person. On the contrary, phenomenology aims to break down the line of demarcation between objectivist ontology and idealist subjectivity [7]. Along these lines, according to Scheler, the person is a concrete essential unit: the foundation of the multiplicity of acts realised by each of us. However, the person is not identified with the mere summation of her/his acts nor with the experience of those acts, because her/his way of being is *to live each act*: we cannot find the person in the already lived acts—[8: 856]. This is part of a more general distancing from Empiricist tradition and its heirs,^3^ such as Ernst Mach and Henri Bergson. As Scheler writes:

Apart from other errors which one can find in these theories, the specific formations of unities of things in the natural view of the world are obviously confounded with the essence of the form of unities: thingness. Of course, one can avail oneself of values in explicating the formation of units of things, but not in explicating thingness [1: 21].

We can, for example, possess with full evidence the beauty of a poem or a painting without being able to say to which factors this value is attached, e.g., color, design, composition, rhythm, musical characteristics, speech-values, picture-values, etc. [1: 196].

As a consequence, the person is not memory:

(…) we can possess with full evidence the values of the object without its being given to us with the same degree of evidence or with the same degree of fullness in its “meaning,” and that we can do this independent of the sphere of remembering [ibid.].

This seems interesting from a juridical point of view, otherwise every technical device with a storage memory would be defined a “person”. We will return to this point in the conclusion.

### Empirical Person Vs. Transcendental Ego

Scheler then goes on to demonstrate that the person is not the “Ego”. Interestingly, Scheler reaches this conclusion after a linguistic analysis of terms such as “ego”, whose meaning is opposed to a “thou” and to the “other world” [1: 389]. On the contrary, for Scheler, a person need not be opposed to another. In a Kantian perspective, the Ego of transcendental apperception is not a correlate that is added to the unity of the object; its unity and identity constitute the condition of the unity and identity of the object. Instead, according to Scheler, a person includes the world, as an objective correlate of experience. This is an application of a fundamental assumption of the realist phenomenology embraced by Scheler: every experience has an objective correlate. For example, if a number of people declare they have shared a telepathic experience, this implies that telepathy has an objective correlate, which can be scientifically analysed by phenomenology, as the phenomenologist Gerda Walther did [9]**.**

Therefore, each individual person carries her/his own individual world and personal truth, not because of relativism (Scheler’s research program in ethics is to oppose relativism) but on account of the essential connection between person and world. On this point Husserl and Scheler agree: “The ego is the constituting source and the person as the fully concrete agent in a social world (…) The person is the focus of moral regard and the bearer of rights” [10]. Even after the “transcendental turn” in his researches, Husserl considered valid the distinction between *transcendental ego* and *person:*

Above all, however, it is versus the empirical subject, in its generality and its unity, that the *“person”* is to be delimited *in the specific sense:* the subject of acts which are to be judged from the standpoint of *reason,* the subject that is *“self-responsible,”* the subject that is free or in bondage, unfree. [11: 269].

The person is the subject equipped with the consciousness of the free “I can”, and not with the mere consciousness that “it will come”, “it will happen”.

However, while Scheler and Husserl seem to agree on the notion of person, they disagree on the very possibility of a transcendental Ego. In Scheler, the notion of person clearly *substitutes* the notion of transcendental Ego. According to Scheler, identity is in the object: objects provide the sufficient, intuitive basis for the law of identity (A = A), and there is not a “condition” to it borrowed from an “Ego”. While in a transcendental perspective the relation between Ego and Object given in the act is unilateral, phenomenological realism describes it as a mutual *one*. From this point of view, Ego can be represented as an object linked to a second object by the act, while the act in itself cannot be represented as an object [1: 374]. Scheler describes this mutual relation as a “Copernican turn”: world-being is a “condition” of the *cogitare* [1: 376].

### Person as a Linguistic Construction

According to several legal systems, some categories of people can be killed without this act constituting a *murder*: slaves, embryos, sons and daughters, wives, enemies in war. In these cases, “there was no givenness (or legal recognition) of the personality of the killed” [1: 315]. These categories were not considered persons, but “animated bodies (in feeling)” [ibid.]: their personality was not given in terms of human individuals, but in terms of units like a master, or a family, tribe, gens, state.

An interesting example, from a semiotic point of view, is Italian law No. 194 of 22 May 1978 on the social protection of motherhood and the voluntary termination of pregnancy [12]. The “non-personality” of the unborn is a meaning resulting from a careful textual strategy which avoids referring to “it” or naming “it”. The Law designs the relations between some actors: counselling centres (10 occurrences); physicians (20 occurrences); women (61 occurrences). The term “abortion” is used only in a dysphoric acceptation (“to prevent abortion from being used for purposes of birth control”; “crime of abortion”) and is substituted by “termination of pregnancy” (35 occurrences), which—in the terms used by structural semantics—underlines a terminative aspectual value. The term “abortion procedures” is used to translate the Italian expression “procedure abortive” in the context of Art. 14, since women are obliged to be informed about them. We also find the “father of the conceptus” (4 occurrences), who cannot of course legally be regarded as a fully-fledged father and is involved only “where the woman consents” (art. 5). Thus, the “father of the conceptus” cannot be considered an actant in the main narrative program and merely plays a role in what Greimas calls an *annex* narrative program [4]. The term “fetus” is used only in two contexts: first, in relation to “abnormalities or malformations”, in a dysphoric acceptation (2 occurrences). In these cases, the English term “fetus” translates the Italian term “nascituro”, which is used quite paradoxically, seeing that it means “someone who is about to be born.” Second (2 occurrences, Art. 7), “Where it is possible that the fetus may be viable”, i.e. in relation to *life*. In this case, the law obliges the doctor to take any appropriate action to save the life of the “fetus”. In all other contexts the use of this term is not necessary. For example:

The physician performing the pregnancy termination shall be required to supply the woman with information and instructions on birth control and to acquaint her with the abortion procedures [12, Art. 14].

The sentence above is well-formed from a syntactic point of view. Other *actants* of the abortion procedures do not need to be specified. According to Luc Boltanski [13] doctors at the ultrasound test draw a similar linguistic distinction between the fetus “adopted” by the parents and the accidental one, which will not be the object of a life project.

According to Scheler, the non-personal “animated bodies” do not embody a “complex of values” that are associated to murder. As the example of the Italian law shows, the attribution of a personal or non-personal value to an entity is mainly a linguistic construction. Furthermore, some of the “depersonalised” entities, such as slaves or war enemies, obviously have a conscience. For this reason, conscience cannot be identified with the notion of personality. The person is not reducible to consciousness, or the object of inner perception, since loving, hating, feeling, willing, have their meaning in the union of the person with a body [1: 392]. For Scheler, the *person* is invariant regardless of “changes in our psychic life”:

What psychiatry says about so-called changes in character in certain psychic illnesses can never pertain to the *person* of the other, even in the most severe cases (e.g., paralysis). It is only the givenness-to-others of his person that disappears. In severe cases we can only say that /his person was made *invisible* by the illness and that a judgment about his *person* is therefore no longer possible [1: 485].

It is worth noting that this position challenges *consciousness-based* bioethical distinctions between person and non-person, according to which the latter, e.g. the embryo or the patient in a vegetative state, can be killed. At the same time, Scheler displays extraordinary sensitivity toward mental illness, ahead of his time. On a similar line, it is known how Husserl considered as persons, at least in a broad acceptation, “abnormal variants of humanness”, such as brutes and some kinds of animals [14: 126]. Nowadays, Husserl’s choice of world sounds poor, but his stance was surely a progressive one when considering the National-Socialist theories of the time. On the subject of animal rights, Scheler grants intelligence, associative memory, and an “essential connection between a consciousness and a lived body” to higher animals [1: 472, n. 104]. Animals feel values (e.g. agreeable/disagreeable; useful/harmful) [1: 266]. However, according to him, “moral facts” cannot be found in nature [1: 164] and we do not expect obedience to moral law on the part of animals [1: 237].

### The Narrative Features of Scheler’s Person

To draw some semiotic conclusions from the above paragraphs, we can identify Scheler’s person as an anthropomorphic actor who embodies the actantial function of a qualified subject. According to Scheler, the person is not reducible to an abstract actor, a human faculty such as “reason”, or “memory”. Thus, the person must be described as a concrete actor. As regards the actantial role, the person must be, at least potentially, capable of action: thus, it is not sufficient to be an object in someone else’s action program to be considered a person: the actor must embody the function of a qualified subject, i.e. a subject in conjunction with the modal value of *knowledge and power.*

### Are There Collective Persons?

There is one last problem related to the description of phenomenological persons as actors: are there collective persons? Law grants the *legal person* (in Latin: *persona ficta*) certain rights, duties, and a degree of responsibility, similar to individual persons. Thus, the relation between individual persons and collective actors, such as companies and government agencies, needs to be clarified. According to Scheler, it is possible to define “collective persons” as the various centres of experience in an endless totality of living with one another [1: 520]. According to this view, every person experiences being a member of a community of persons, a social unit, described as a sphere of co-responsibility. Husserl agrees on the presence, in the world, of “personalities of a higher order” (state, church) [14: 77].

However, according to Edith Stein, when identifying communities of life as a collective person, Scheler encounters a difficulty in relating individual responsibility and freedom to the collective responsibility of the community [15: 276]. Stein notes that in an empirical community, not every member is a free and fully responsible person, and speculates about a collective person whose members are not responsible persons:

If the community no longer contains any free persons, or none who build up the community with their personal living, then it is certainly no longer capable of any goal-setting. Then the question is, what is left to the community at all. Not a shred of responsibility, it seems to me, first of all. You can no longer talk of responsibility in any sense in a community in which no single person is responsible. There is no free acting here, no more free self-formation, but only an impulsive doing that no longer can be considered as bearer of any responsibility [15: 276].

To solve this difficulty, we will now resume Edith Stein’s analysis of the *nexus* between individual persons and their consequences in terms of philosophy of law.

## Person and Community in Edith Stein

Edith Stein was Husserl’s scholar (1913) and private assistant (1916–1918). She also attended the lectures Max Scheler gave to the Göttingen Philosophical Society in 1913 [10]. She dedicated her doctoral dissertation to empathy, a kernel problem in phenomenology [16]. Both Husserl and Max Scheler [17] investigated forms of sympathy, empathy, and love to explain how we grant the status of subject to other people, avoiding the risk of solipsism related to the analysis of the intentional relation between one’s consciousness and the world. Later on, Edith Stein proposed an interesting phenomenological analysis of grief, aimed at studying the relationship between individual and community [15]. In particular, she considered the following example: the army unit in which I am serving is grieving over the loss of its leader.

Thus everyone has grief that is individually *his or hers:* even though it is legitimate to say, on the other hand, that they all feel “the same” grief. This “selfsameness” has significance that merits precise exposition. The grief is quite a private content that I feel, but it is *not only* that. It has a sense, and by virtue of that sense it claims to count for something lying beyond the private experiencing, something subsisting objectively, through which it is rationally substantiated. In our sense the objective item to which the grief applies according to its sense is the loss of the leader. Thus the correlate of experience is the same for everyone who participates in it. [15, 135].

Again, we find the ground assumption of realist phenomenology, according to which each experience corresponds to an objective correlate. Referentialist semiotic theories of the period play a role in the identification of the correlate of experience: individual grief has a meaning if and only if this meaning is related to an ontological counterpart, a *state-of-affairs* or a situation, by a judgement. Husserl discusses judgements in [6, 5th and 6th research], merging Brentano’s and Bolzano’s views. To summarise his position, judgement is built up on perception, imagination, memory, etc. articulating and specifying a state-of-affairs which is linked by the rays of a sort of geometrical projection. It implies an act of will, a stance, and—when sedimented—it can become a belief and a conviction. Thus, judgement can be life-changing: if, on the one hand, we are conscious of our own life as endlessly flowing [18: 145], on the other it is a continual striving for a judicative decision and the establishment of passed judgements [18: 100], a striving after intuition that realises the intended self [18: 146].

### Rights from Empathy

Edith Stein’s analysis of grief, presented above, proves that, as an entity, the person presupposes a relationship with other people: being a person does not mean being unique and unrepeatable but being in *mutual* empathic recognition. In structural terms, we can resume the analysis of the lexemes “person” and “community” as follows:

Person = /individual/ + /subject/

Community = /collective/ + /subject/

In the formulae??, /individual/, /collective/, /subject/, and /object/ are values in the acceptation of structural semantics [19]. In this perspective, the Other is recognised as person by the community:

Other = /individual/ + /subject/

The intersubjective relationships established by empathy enable us to recognize the Other as a person, therefore as a bearer of rights and duties; the intersubjective circularity of this recognition is the basis for universal and unconditional reciprocity, through the establishment of the legal system.

### Inferior Human Beings with No Rights

There is an important difference between Husserl’s transcendental point of view and Stein’s empirical one: according to Husserl, if empathy characterizes the transcendental Ego, i.e. if it is the condition of possibility of the Ego, we are always in a situation of harmony in the community of Egos. This would lead to the foundation of one and only one State, and one and only one legal system, based on human rights. On the other hand, Stein’s empirical analysis does not admit the method of the “epochè”, i.e. the absence of commitment about the existence of the world aimed at experiencing the transcendental features of the Ego. If empathy is a transcendental feature, then every human being should always be in a relation of empathy with other humans: it is the notion of transcendental intersubjectivity. On the contrary, empirical experience shows many situations in which the relation between society and the Other is merely instrumental and the latter is just an object:

Other = /individual/ + /object/

As an example, let us think of the inferior status accorded by many legal systems to different categories of persons, like immigrants and their children in current Italian law, or Jews in Nazi Germany. A case in point is Pierre L. van den Berghe’s notion of *herrenvolk democracy:*

A dual political system in which the ruling ethnic group maintains a representative government with a façade of formal democracy for itself and rules the rest of the population as colonial subjects [20: 165].

The author extended this notion from South Africa to the United States and Israel, but current Italian law on immigration can be considered a special instance, as it does not grant certain rights to particular categories of immigrants, for example, differentiating healthcare assistance.

### Community, Association, Mass

In a controversy with Martin Heidegger, Husserl interpreted instrumentality in human relations as part of a crisis in European culture [21]; on the contrary, Edith Stein considers the social unit based on instrumental relations as a mere empirical possibility. She defines as “association” the social unity in which the individuals gathered together regard one another reciprocally as objects. However, while associations are nevertheless collective subjects, Edith Stein acutely observed a new political phenomenon of her times: the rise of the masses and their manipulation by charismatic leaders. Stein agrees with Scheler [1] on the fact that a mass is constituted by passive contagion and involuntary imitation. Unlike communities and associations,

The individuals who are gathered together into the mass are generally not oriented toward one another (…). Their sentient life occurs only isomorphically with that of the others, who are joined with them into a unity by being gathered spatially, and, to be sure, *as a result of* being gathered together. (…). [15: 241].

Her views on the passivity of masses are influenced by Simmel [22]. According to Edith Stein, the consistency in the behaviour of the mass (…) lends it its character of “collective objectivity” [15: 243]. Thus, we can say:

Mass = /collective/ + /object/

## Discussion

To resume our research, on the basis of the semiotic interpretation of the phenomenological notion of person in Max Scheler and Edith Stein presented above, we can define the person as an individual, anthropomorphic actor who embodies the actantial function of a qualified subject. However, the notion of person is also a relational feature: in order to be a person, any individual must empathically recognize other persons and be recognized by them. This happens in a particular social unit, the community. The intersubjective circularity of this recognition of rights and duties is the basis for the establishment of the legal system. Nonetheless, while empathy tends to establish universal legal systems based on human rights, other kinds of relations between individuals are possible: instrumental, programmed relationships, and contagion. Different societies and laws are formed by combining the semiotic categories Collective/Individual and Subject/Object. They also underlie the inclusion and the exclusion of human groups, giving rise to hierarchies of humans. Table 1 resumes the relation between the notion of person as an individual actor with the role of qualified subject and the collective actors with which she/he is always relationally entangled.

**Table 1:** Social unities according to Edith Stein

	Community	Association	Mass
Is a collective …	Subject	Subject	Object
Orienting function	Empathy	Instrumental relationships	Contagion
Relation between individuals	Subject–Subject (person)	Subject–Object	Absence of relation

### Case History: Artificial Intelligence

It is possible to attribute personal or non-personal semantic values to an algorithm or to a set of algorithms. This is made possible by a semiotic strategy similar to the one we saw in paragraph 1.3. For example, let us compare two definitions, retrieved from websites:

In statistics, linear regression is a linear approach to modelling the relationship between a scalar response and one or more explanatory variables (also known as dependent and independent variables) [23].

Machine learning is the ability for a computer to output or do something that it was not programmed to do. While machine learning emphasizes making predictions about the future, artificial intelligence typically concentrates on programming computers to make decisions. If you use an intelligent program that involves human-like behaviour, it can be artificial intelligence. However, if the parameters are not automatically learned (or derived) from data, it is not machine learning. [24].

The two definitions are broadly equivalent: the linear regression algorithm is widely used in supervised machine learning [25: 277]. However, nobody would ascribe a legal personality to a statistic approach linking some variables to an approximating function. On the contrary, the second definition attributes human features to the algorithm: it is not programmed; it learns from data; it is intelligent; it predicts the future; it behaves like a human. The humanity of the algorithm is semiotically constructed through texts and images representing brains, heads, sparkling neurons.

Let us analyse a juridical text, the European Parliament resolution of 16 February 2017 with recommendations to the Commission on Civil Law Rules on Robotics (2015/2103(INL) [26] and its summary [27]. The document asks the Commission to explore the implications of different topics. Among the others:

creating a specific legal status for robots in the long run, so that at least the most sophisticated autonomous robots could be established as having the status of electronic persons responsible for making good any damage they may cause [27].

In the summary, robots are defined as “smart” (3 occurrences). According to the document, the features that define a smart robot are:

- the acquisition of autonomy through sensors and/or by exchanging data with its environment (inter-connectivity) and the trading and analysing of those data;
- self-learning from experience and by interaction (optional criterion);
- at least a minor physical support;
- the adaptation of its behaviour and actions to the environment;
- absence of life in the biological sense [26: 1]

According to the Parliament, “special attention should be paid to the possible development of an emotional connection between humans and robots ‒ particularly in vulnerable groups (children, the elderly and people with disabilities).”

After the adoption of the text, a number of Artificial Intelligence and Robotics experts, industry leaders, and experts in law, medicine and ethics wrote an open letter to the European Commission [28]. According to this letter, the Parliament’s resolution is “distorted by Science-Fiction and a few recent sensational press announcements”. Nevertheless, the possibility of creating a status of “electronic person” on the model of the status of “legal person” is still the object of studies commissioned by the European Parliament [29].

If we adopt Scheler’s point of view, personhood cannot be identified with any of the features of the new robots (smartness, learning skills, adaptation to the environment), since personhood is the basis that assures their unity. However, this essentialist point of view gives us no indication on how to prove the existence of an objective correlate of a supposed electronic personhood. Is there a ghost in the shell?

Adopting Edith Stein’s perspective can be interesting, since the document indicates a possible intersubjective relation between the robot and human beings (“emotional connections”). Thus, some humans could consider robots as “persons” when interacting, working, or playing with them: as individual subjects belonging to the collective subject “community” on the basis of mutual empathic recognition. A similar argument could be advanced to extend personhood to specific animals, in order to grant them some rights.

On the other hand, the Parliament proposes a second intersubjective reason to extend the notion of personhood to robots: the reparation of the damage they can cause. In this case, the relation between humans (subject) and robots (object) is merely instrumental.

### Further Developments

The notion of person we receive from the phenomenological debate seems useful in evaluating interesting new legal problems, such as the responsibility of machine learning algorithms and artificial intelligence. A computer has a memory and can keep track of every performed “act”. I have placed the expression in inverted commas, since this memory seems quite different from that of humans. For example, while a man can be considered responsible for forgetting, a computer cannot. There is obviously an intentional element in human remembering, which does not allow us to consider it a mechanical process. Can a refined form of statistical calculation performed by a machine be considered “responsible” for ethical acts and judgements? The question does not appear to be a rhetorical one: the “unbiased” decisions of these machines are used for probation, or to flag hate speeches in social networks for forensic purposes, thus substituting human moral judgement. The same intentional behaviour seems to be the rationale for moral responsibility, and to express a limit to the possibility of identifying human beings and animals in relation to their capacity of expressing preferences—which are, in some cases, very broad—and taking a role in social life.

With the help of the notion of enunciation, let us remember that a number of non-personal, non-human, and even non-figurative actors can embody the actantial role of subject. According to Benveniste, the identification between non-personal and non-subjective is misleading:

Here again we come up against the question of the impersonals, an old problem and a sterile debate as long as we persist in confusing ‘person’ and ‘subject.’ (…). The ‘third person’ must not, therefore, be imagined as a person suited to depersonalization. There is no apheresis of the person; it is exactly the non-person, which possesses as its sign the absence of that which specifically qualifies the ‘I’ and the ‘you.’ Because it does not imply any person, it can take any subject whatsoever or no subject, and this subject, expressed or not, is never posited as a ‘person.’ This subject only adds *in apposition* a precision judged necessary for the understanding of the content, not for the determination of the form. Hence *volat avis* does not mean 'the bird flies,' but 'it flies (scil.) the bird.' The form *volat* would be enough in itself and, although it is nonpersonal, includes the grammatical notion of subject [30: 199].

I should like to emphasize this. Phenomenology is the last humanistic philosophy of the 20th Century; the subsequent psychoanalytic, structuralist, post-structuralist, constructivist, relativist and various feminist theories challenged the notion of subject and attempted to disassemble it. Phenomenological notions of person are essentialist: the person is not a sum of acts or memories; on the contrary, it is the necessary condition for their unity. Furthermore, it belongs to the essence of the person to exist and to live solely in the execution of intentional acts [1: 390]. However, in Edith Stein’s reflections on empathy and society, a more relational notion is displayed: it takes a person to recognise a person. Thus, intersubjectivity seems the real premise to every phenomenological inquiry, as in the Cartesian Meditations [14]. Intersubjective, social relations founded on empathy provide a possible explanation to the issue we presented at the beginning of the present paper: how can law-making processes arise from a collective experience of injustice? To make this possible, the content of the individual experience of injustice must be shareable even if it is not directly experienced. This way it is the foundation of the formation of a collective social actor. From a semiotic point of view, we can substitute “shareable” with “meaningful”, identifying in the contagion the mechanism of the communication channel. In fact, the phenomenological analysis of social unities, presented above, shows an affinity to the semiotic analysis of the interaction proposed by Eric Landowski on contagion [31: 115]. According to Conrado Moreira Mendes,

Contagion operates without the mediation of value-objects; it is not the logic of junction anymore, but that of union, which governs contagion. It still does not operate on the cognitive plane, but on the sensible plane. Landowski refers to interaction by contagion, taking laughter as an example: a subject that is infected by the laughter of the other without any transfer of object-value [32: 141].

The absence of value-objects can be puzzling: according to Scheler, moral values are invested in actions. But these cannot be interpreted as value-objects since they are not exchange-objects. However, “absence of objects” does not necessarily mean “immediacy”: Landowski recognises a form of contagion mediated by *vectors—*for example, music [31: 185].

On the contrary, the instrumental relationships described by Edith Stein could be considered a case of programmed relations [33]. According to Paolo Demuru,

In programming, subjects regularly follow the narrative paths ruled by principles of physical causality and/or social coercion. This is the realm of routine and habits in which, as Greimas had already pointed out in *De l’Imperfection* [34], there lies a tendency to the insignificance and exploitation of meaning [35: 87].

The dialectic between community and society or between universalization and exclusion [36: 29–44] can be seen, in socio-semiotic terms, as the opposition between adjustment and programming, meaningfulness and insignificance, between make happen and make feel [35: 88] and it explains why it is not possible to speak about a progressive, cosmopolitan and universalising evolution of law toward inclusive citizenships. Dershowitz’s perspective comes up against an insuperable boundary: one of the typical manifestations of human nature is the negation of its own generality [37: chapter 2]. Since it is based on the intersubjective mutual recognition of belonging to a community, personhood can be extended beyond the limits of the human (as in the case of legal and “electronic” persons); however, at the same time, some “human entities” must be excluded from the category.

## Footnotes

1 A compendium of classical philosophy is outside the scope of this paper. We recall Plotinus’s Enneads, fifth book, which merges Aristotelian and Platonic tradition and which deeply influenced later Christian philosophy by presenting the doctrine of the hypostases [5].

2 See for example Thomas Aquinas, *Summa Theologiae,* first part, question 76, Article 1: “the intellect which is the principle of intellectual operation is the form of the human body. For that whereby primarily anything acts is a form of the thing to which the act is to be attributed: for instance, that whereby a body is primarily healed is health, and that whereby the soul knows primarily is knowledge; hence health is a form of the body, and knowledge is a form of the soul. The reason is because nothing acts except so far as it is in act; wherefore a thing acts by that whereby it is in act. Now it is clear that the first thing by which the body lives is the soul. And as life appears through various operations in different degrees of living things, that whereby we primarily perform each of all these vital actions is the soul. For the soul is the primary principle of our nourishment, sensation, and local movement; and likewise of our understanding. Therefore this principle by which we primarily understand, whether it be called the intellect or the intellectual soul, is the form of the body. This is the demonstration used by Aristotle (De Anima ii, 2)” [6].

3 I am in debt to Gabriele Vissio, who called my attention to this point.
